# Recent advances in lung transplantation

**DOI:** 10.12688/f1000research.15393.1

**Published:** 2018-10-23

**Authors:** Keith C Meyer

**Affiliations:** 1 UW Lung Transplant & Advanced Pulmonary Disease Program, Section of Allergy, Pulmonary and Critical Care Medicine, Department of Medicine, University of Wisconsin School of Medicine and Public Health, Madison, WI, USA

**Keywords:** lung transplantation

## Abstract

Lung transplantation can improve quality of life and prolong survival for individuals with end-stage lung disease, and many advances in the realms of both basic science and clinical research aspects of lung transplantation have emerged over the past few decades. However, many challenges must yet be overcome to increase post-transplant survival. These include successfully bridging patients to transplant, expanding the lung donor pool, inducing tolerance, and preventing a myriad of post-transplant complications that include primary graft dysfunction, forms of cellular and antibody-mediated rejection, chronic lung allograft dysfunction, and infections. The goal of this manuscript is to review salient recent and evolving advances in the field of lung transplantation.

## Introduction

Lung transplantation has become an accepted treatment option for patients with various forms of advanced, progressive lung disease that does not respond to non-transplant therapies. Although early post-transplant survival has gradually improved with the implementation of advances in surgical techniques and peri-operative management, many early complications, such as surgical complications or primary graft dysfunction (PGD), threaten lung allograft function and viability, and various complications with delayed onset, such as chronic lung allograft dysfunction (CLAD) or opportunistic infection, continue to significantly impact recipient survival and long-term outcomes. Although an increasing number of patients considered for lung transplantation are extremely ill and require ventilator and/or extracorporeal life support (ECLS) as a bridge to transplant, the last 10 years have seen developments in management pre- and post-transplant that have yielded modestly improved outcomes, including the establishment of revised guidelines for selecting candidates, enhanced surgical techniques, improved methods for donor lung preservation, advances in suppressing and treating allograft rejection, the development of prophylaxis protocols to decrease the risk of opportunistic infection, implementing more effective therapies for treating infectious complications, and the development of a clinical practice guideline concerning the diagnosis and treatment of bronchiolitis obliterans syndrome (BOS) and other forms of CLAD.

## Candidate selection, timing of referral, waitlist placement, and lung allocation

A consensus update to previously published documents that addressed the selection of lung transplant candidates was published by Weill *et al*. ^1^ in 2015 under the auspices of the International Society for Heart and Lung Transplantation (ISHLT). A notable change in the eligibility criteria was the recommendation that age by itself should not be considered a contraindication to transplantation with the caveat that patients over 75 years old are unlikely to be candidates, and age over 65 years, if associated with low physiologic reserve and/or other relative contraindications, should remain a relative contraindication. In addition to providing absolute and relative contraindications to lung transplantation, recommendations concerning disease-specific timing of referral and waiting list placement, the use of mechanical and/or ventilator support as a bridge to transplant, special surgical considerations, and other issues were discussed in the document. Of note, the issues of sarcopenia and frailty, which evolving literature has identified as having an important impact on lung transplant outcomes ^2– 4^, were not recognized in the guideline as relative contraindications.

The lung allocation score (LAS) was implemented in 2005 to provide a system for donor lung allocation in the US based on urgency and benefit rather than time accrued on the waiting list, and use of the LAS has reduced the risk of death without transplant for waitlisted patients while improving the effectiveness of organ allocation and transplantation by increasing the number of transplants and shifting the distribution of donor lungs to patients who are more likely to die on the waiting list ^5, 6^. However, outcomes have been reported to remain somewhat worse for recipients with very high LAS scores ^7– 9^. Nonetheless, it was recently suggested that recipients with higher LAS values and specific transplant indications, such as idiopathic pulmonary fibrosis (IPF) or cystic fibrosis (CF), benefit the most from lung transplantation in terms of survival ^10^, and recipients with the transplant indication of pulmonary fibrosis and very high LAS values can achieve prolonged post-transplant survival that does not differ significantly from that seen in those with lower LAS values ^11^. A recent trends analysis that used the United Network for Organ Sharing (UNOS) database found that recipients with LAS values in the upper quartile transplanted in the more recent era of 1 January 2012 to 31 March 2014 had significantly improved mortality at 30 days and 1 year versus those in earlier eras ^12^. The success of the LAS system in the US has led to its adoption in non-US countries (Germany in 2011 and the Netherlands in 2014) ^13^. Nonetheless, there remains a need to further improve the lung allocation process to decrease the risk of poor outcomes and associated increased costs.

## Expanding the donor pool

Donation after brain death (DBD) donors represent the traditional and largest source of donor lungs, and relaxing ideal criteria to allow the use of marginal donor organs that meet extended criteria has increased the numbers of lungs available for transplant ^14^. Another source of donor lungs that is increasingly being used to help meet the persistent shortage of donor lungs is that of donation after circulatory death (DCD) donors, a source that remains considerably underutilized but could significantly increase the pool of donor lungs, especially when coupled with donor lung resuscitation and evaluation using *ex vivo* lung perfusion (EVLP). Chancellor *et al*. ^15^ performed a chart review at their quaternary care center and identified 85 patients (out of 512 who did not have any evidence of lung pathology) over a one-year time period who met criteria that qualified them as potential DCD lung donors. Costa *et al*. ^16^ compared outcomes for 46 DCD lung transplants (lungs were suitable for use from 44 out of 73 DCD donors) to those of 237 transplants from DBD donors and found that rates of PGD and survival were equivalent for DCD versus DBD transplants. Similarly, a multicenter study performed in the Netherlands did not identify significant differences in PGD, freedom from CLAD, or overall survival for 130 DCD recipients versus a matched cohort of 130 DBD lung recipients ^17^, and Ruttens *et al*. ^18^ retrospectively reported a similar experience from Belgium in which no significant differences in time of ventilator support, length of intensive care unit or hospital stay, PGD score, freedom from CLAD, or overall survival for 59 DCD as compared to 331 DBD recipients were identified.

## Bridging to transplantation

Outcomes for lung recipients on either mechanical ventilation or devices that provide ECLS pre-transplant are generally worse than outcomes for recipients who do not require such support ^11, 19– 20^. However, the use of ECLS as a bridge to transplant for patients with severe respiratory failure has gradually increased ^1, 21– 23^, and ECLS may provide the only means of keeping a patient alive and can also be used to support recipients through the transplant procedure ^24– 27^. An evolving experience with ambulatory ECMO has demonstrated that this approach can be used to avoid deconditioning and allow patients to be ambulatory while awaiting organ offers and transplantation ^28– 32^, and using ambulatory ECMO has recently been shown to have economic advantages over non-ambulatory ECMO ^33^. Recent queries of the UNOS database have shown that while the use of ECMO had an adverse impact on survival for older patients, a negative effect was not demonstrated for a subgroup of younger patients under 40 years of age ^34^, and when used in high-volume transplant centers, ECMO use did not appear to increase the risk of post-transplant mortality ^35^.

## *Ex vivo* lung perfusion

EVLP allows explanted donor lungs to be perfused and ventilated while being evaluated and reconditioned prior to implantation ^36, 37^. While donor lungs are supported by EVLP, the physiologic status of lungs with marginal function and/or evidence of significant injury can be observed and potentially improved ^36– 42^, and if an unacceptable degree of persistent dysfunction is identified, transplantation of inadequately functioning donor lungs can be avoided. EVLP allows fluid to be drawn from extravascular compartments in edematous lungs such that gas exchange can be improved and marginal lungs rendered usable, and anti-inflammatory cytokines or mesenchymal stem cells (MSCs) can be employed to accelerate repair after injury, encourage recovery in intercellular alveolar epithelial tight junctions, improve oxygenation, and decrease vascular resistance ^43– 46^. In addition, antibiotics can be administered to reduce or eradicate donor lung infection ^47^, gastric acid-injured donor lungs can be potentially salvaged ^48^, and perfusate can be analyzed to detect biomarkers that predict high risk for early allograft dysfunction ^49, 50^. Finally, Yeung *et al*. ^51^ recently demonstrated that acellular normothermic EVLP was associated with a progressive decline in cell-specific leukocyte gene expression over a 12-hour period, which suggested that this technique can potentially interrupt an inflammatory response via washout of intravascular leukocytes, and metabolomic characteristics of EVLP perfusate may serve as biomarkers that can predict PGD risk and improve the selection of marginal lungs that can be used for transplant ^49^. EVLP is likely to significantly expand the usable donor pool as donor lungs are provided to both large- and smaller-volume institutions from EVLP hubs ^52– 56^. Two EVLP devices have been approved by the Food and Drug Administration for clinical use, and a number of clinical trials evaluating the use of EVLP to evaluate and potentially recondition marginal donor lungs are currently in progress.

## Primary graft dysfunction

PGD is defined as lung injury that occurs within the first 72 hours following lung implantation as reflected by the appearance of diffuse allograft edema/infiltration on chest radiograph, and PGD severity is staged according to PaO _2_ (arterial oxygen tension in mmHg)/FiO _2_ (fraction of inspired oxygen in inhaled gas) ratios with >300 for mild (stage 1), 200–300 for moderate (stage 2), and <200 for severe (stage 3) ^57^. Notably, analysis of data from the Lung Transplant Outcomes Group multicenter recipient cohort (1,179 subjects from 10 lung transplant centers) did not detect mortality differences between patients with no (stage 0) and mild (stage 1) PGD, and the authors concluded that the PGD consensus definition could be simplified by combining stages 0 and 1 ^58^.

Nearly one-third of lung transplant recipients have been reported to develop stage 3 PGD ^57^, and while a number of markers have been identified that correlate with increased risk of high-stage PGD ^59, 60^, interventions other than supportive care have been relatively ineffective in preventing or treating PGD ^61, 62^. Risk factors for developing PGD include both donor- and recipient-related factors, surgical factors at the time of transplantation, epithelial injury, endothelial injury and activation, and triggering of both innate and adaptive immune responses ^60^. A key early event in PGD pathogenesis is the translocation of neutrophils from circulating blood to the interstitium and air spaces in response to chemotaxins such as damage-associated molecular pattern (DAMP) moieties or CXCL8 that are released as a consequence of ischemia-reperfusion injury ^61^. Various pro-inflammatory cytokines, reactive oxygen intermediates, and proteolytic enzymes represent prominent mediators of tissue disruption in the reperfused, ischemia-damaged lung, and other cell types, such as lung macrophages and lymphocytes, also play a role in the acute lung injury and the loss of epithelial integrity and resultant interstitial edema that occurs in PGD.

A number of collaborative studies performed and published by the Lung Transplant Outcomes Group have provided additional insights into PGD risk factors and pathogenesis. Notable observations include the association of a genetic variant, Toll-interacting protein rs3168046 (TOLLIP), with clinical PGD and with higher circulating plasminogen activator inhibitor-1 (PAI-1), which has been previously identified as a quantitative PGD lung injury biomarker ^62^. Other recent studies have identified a pathogenic role of neutrophil extracellular traps ^63^, a decreased risk of stage 3 PGD when allografts are oversized as reflected by predicted donor and recipient total lung capacity estimations ^64^, variations in oxidant stress gene expression and PGD stage 3 risk ^65^, and a role for cell-free hemoglobin in causing oxidative endothelial injury that can increase vascular permeability and contribute to PGD ^66^.

## Immune tolerance

A key goal in lung transplantation is to achieve a state wherein a destructive alloimmune response does not occur when immune responses are not globally suppressed. Mixed chimerism has been shown to allow tolerance to emerge in both murine and large animal models, although mechanisms by which this is achieved differ among species, and sustained chimerism and tolerance have been particularly difficult to achieve in primates ^67^. Of interest, it has been shown that in a mouse model involving primary transplantation of fully major histocompatibility complex (MHC)-mismatched lungs that were subjected to a 72-hour period of costimulatory pathway blockade (peri-operative blockade of B7-CD28 and CD40–CD40 ligand costimulatory pathways), retransplantation of the lungs into T-cell-deficient recipients led to long-term acceptance that could be abrogated if the primary recipients had received anti-CD25 antibodies ^68^. Another murine model study by this group identified CCR7-expressing CD8 ^+^ T cells that interacted with CD11c ^+^ antigen-presenting cells and infiltrated lung allografts as playing a critical role in tolerance induction ^69^. Although immune checkpoint inhibition has been studied fairly extensively in animal models of allogeneic organ transplantation, experience with immune checkpoint inhibitors to modulate allograft tolerance in human organ transplant recipients is limited to anecdotal case reports ^70^ and a phase I/Ib clinical trial in allogeneic stem cell transplantation in Japan ^71^. Advances in understanding immune tolerance and translating such knowledge to clinical application in allogeneic lung transplant represent a key strategy to lessening the risk of PGD, acute rejection, and CLAD. Simultaneous infusion of donor bone-marrow-derived cells at the time of lung transplantation has been associated with a higher incidence of donor-specific hyporeactivity as determined by one-way mixed leukocyte culture as compared to controls ^72^, and long-term lung allograft survival without immunosuppression in a patient who simultaneously received T- and B-cell-depleted bone marrow has been reported ^73^.

## Chronic lung allograft dysfunction

The greatest threat to long-term survival for recipients who survive beyond one year post-transplant is the onset and progression of CLAD ^74– 76^. This syndrome was initially termed bronchiolitis obliterans syndrome (BOS) in the 1990s ^77, 78^ and diagnosed on the basis of a persistent ≥20% decline from best post-transplant forced expiratory volume in one second (FEV1) without other identifiable cause, and BOS was assumed to be a consequence of chronic rejection and the cause of persistent decline in the surrogate marker of FEV1. It subsequently became clear that a multitude of complications can cause such a decline in FEV1 ^74^, and the term CLAD has supplanted the formerly used, overarching term BOS ^79^. Recent analyses of the literature and a consensus revision of terminology have recognized two major CLAD phenotypes, which are obstructive CLAD (BOS) and restrictive CLAD (restrictive allograft syndrome) ^74, 79^. The American Thoracic Society (ATS)/European Respiratory Society (ERS)/ISHLT clinical practice guideline systematically examined available evidence that had accrued in the literature pertaining to BOS/CLAD, and an expert consensus committee provided recommendations for its diagnosis, prevention, and treatment ^74^. Identified risk factors included PGD, various forms of alloimmune rejection (acute cellular rejection, antibody-mediated rejection, and lymphocytic bronchiolitis), infections (viral, bacterial, and fungal), pathologic GER, autoimmunity, and persistent bronchoalveolar lavage (BAL) neutrophilia. Despite the evidence from randomized controlled trials (RCTs) in the prevention and treatment of BOS/CLAD being low or very low in quality, a number of conditional recommendations were made by consensus among task force members following a comprehensive review of available publications ( Table 1).

**Table 1.** International Society for Heart and Lung Transplantation/American Thoracic Society/European Respiratory Society clinical practice guideline recommendations for the prevention and treatment of bronchiolitis obliterans syndrome (BOS)/chronic lung allograft dysfunction ^76^ *.

1. Administer enhanced immunosuppression if acute cellular rejection of stage 2 or higher or lymphocytic bronchiolitis is found on lung biopsy 2. Consider enhanced immunosuppression if minimal acute cellular rejection (stage 1) is found on biopsy 3. Avoid use of long-term, high-dose corticosteroids to treat BOS 4. If BOS develops while patients are on cyclosporine A as their calcineurin inhibitor, consider changing to tacrolimus 5. Consider a trial of azithromycin for the treatment of BOS 6. If significant gastroesophageal reflux is identified in a patient who develops BOS, consider referral to an experienced surgeon for potential fundoplication to prevent reflux 7. Consider referring recipients with end-stage BOS that is refractory to treatment for re-transplantation

*All are conditional recommendations with very low quality of evidence

Restrictive CLAD has a worse prognosis than does obstructive CLAD ^80, 81^, and both phenotypes tend to respond poorly to augmented immunosuppression or other interventions. Initial treatment of CLAD/BOS consists of modifying the maintenance immunosuppressive drug regimen. However, such therapy is generally ineffective for treating progressive CLAD. RCTs have suggested that CLAD can be prevented and/or attenuated by administering azithromycin ^82, 83^, but evidence for other therapies remains relatively lacking ^84^. Benden *et al*. ^85^ systematically examined publications pertaining to salvage therapy for progressive CLAD/BOS and concluded that available data were supportive of a role for extracorporeal photopheresis (ECP) combined with established immunosuppressive regimens, but other approaches (e.g. total lung irradiation and montelukast) had few data to support their therapeutic use.

Predicting risk for developing CLAD has been elusive. Koutsokera *et al*. ^86^ examined outcomes data from French and Swiss transplant cohorts (SysCLAD Consortium) and reported that the best prediction model for early onset CLAD included underlying diagnosis as the indication for transplant, type of induction regimen, maintenance immunosuppression regimen, and whether donor-specific antibodies (DSA) directed against class II HLAs were present at one year post-transplant. Many biomarkers have been associated with CLAD, but none have been validated as clinically useful. Speck *et al*. ^87^ performed a comprehensive literature search that sought to identify potentially useful cytokines (e.g. interleukins, chemokines, interferon-γ, tumor necrosis factor, and transforming growth factor-β) in plasma and/or BAL, but none could be considered suitable as diagnostic markers. More recently, Durand *et al*. ^88^ reported that a threshold increase in CD4 ^+^CD25hiFoxP3 ^+^ T cells at one and six months post-transplant was associated with a two-fold increased risk of developing BOS.

Additional recent publications of interest include those examining the potential utility of magnetic resonance imaging as an adjunct to spirometric measurement of FEV1 to diagnose CLAD and characterize/quantitate CLAD-related change ^87, 89^. Haynes *et al*. ^90^ evaluated whether a HLA-DR restricted effect on BOS/CLAD incidence could be detected in a large, single-center recipient cohort with long-term follow up; the presence of donor HLA-DR15 (which avidly binds α1 chain peptides of collagen V [colV] and is associated with anti-colV autoimmunity) was associated with a significant increase in susceptibility to severe BOS, while donor HLA-DR7 or recipient HLA-DR17 expression was associated with reduced susceptibility.

## Lung microbiome

Investigations of the lung microbiome in health and disease have shown that it varies considerably among disease states ^91, 92^. Bacteria, archaea, fungi, and viruses that comprise specific lung microbiomes are constantly interacting with each other and with host immune responses and defense mechanisms, and lung transplant recipients must adapt to the newly acquired donor microbiome ^93, 94^. Sharma *et al*. ^95^ have reported that microbiome signatures of the mouth, nose, and proximal and distal airways in transplant recipients show site-dependent variation, with the nasal microbiome closely resembling that of the proximal and distal airways; myeloid-derived suppressor cell (MDSC) phenotypes were found to predominate in proximal airways, while a preponderance of pro-inflammatory MDSCs was found in distal airways, and the abundance of distinct bacterial phyla correlated with specific MDSCs in proximal versus distal airways. Mouraux *et al*. ^96^ demonstrated a correlation between the presence of pro-inflammatory bacteria (e.g. *Pseudomonas, Staphylococcus,* and *Corynebacteria*) in BAL and catabolic lung remodeling gene expression; however, when bacteria known to be associated with a healthy, steady-state microbiome (e.g. *Streptococci, Prevotella,* and *Veillonella*) were found in BAL, so was the expression of anabolic remodeling genes. Additional investigation of the transplanted lung microbiome and host–microbiome interactions is needed to provide insights into injury responses and matrix remodeling as well as alloimmune responses, allograft tolerance, and the pathogenesis of CLAD ^94^.

## Stem cells, xenotransplantation, and bioengineering

Although donor-derived MSCs have been identified as potentially playing a significant role in bronchiolar fibrosis in BOS/CLAD ^97– 99^, MSCs may also be capable of inhibiting immune cell function (e.g. T cells, B cells, NK cells, and dendritic cells) and cytokine secretion ^100, 101^. Numerous studies in animal models have demonstrated stem cells’ ability to lessen injury and inflammation ^102, 103^, and stem cell therapies may blunt acute ischemia-reperfusion injury and fibrotic responses to lung injury, thereby preventing or limiting the severity of PGD ^103^. In addition, MSCs may have the capacity to encourage alveolar fluid clearance in lungs that otherwise would not be suitable for transplantation because of edema and impaired gas exchange ^104^, and early experience using allogeneic bone-marrow-derived MSCs in human lung transplant recipients with deteriorating CLAD showed slowing of the decline in FEV1 and supports the feasibility of using such therapies in patients with advanced CLAD ^105^.

Xenotransplantation could be an alternative approach to allogeneic lung transplantation or act as a bridge to allotransplantation by extending the time period for a human donor–recipient match to be found with subsequent replacement of the xenograft when a human donor lung becomes available ^106, 107^. Extensive genetic engineering of pigs as a lung donor source is ongoing, but genomic restructuring is needed for the prevention of the acute thrombotic and severe inflammatory reactions seen in various models such as pig and primate xenotransplantation ^106, 108, 109^. Ongoing efforts to knock out xenoantigens to avoid triggering coagulation and inflammatory cascades and complement activation in partnership with pharmacologic manipulation could one day lead to the transplantation of pig lungs to humans ^110^.

Although bioartificial lung grafts derived by re-cellularizing an intact whole lung extracellular matrix with stem or progenitor cells have been successfully placed orthotopically in animal models and can provide gas exchange, delayed onset of inflammation and consolidation has eventually led to loss of function ^111, 112^. Research is ongoing to determine whether bioengineered lungs that have been adequately re-cellularized with appropriately differentiating stem cells that can replace or repopulate the over 40 various cell types in their usual anatomic sites can sustain prolonged function ^113^.

## Summary and conclusions

Many key advances ( Table 2) have occurred over the past few years that are likely to lead to increased donor organ availability and improve both short- and long-term outcomes in lung transplantation. However, many key questions remain ( Table 3), and studies in animal models as well as multi-center clinical investigations are needed to address these issues. Systematic literature reviews and evidence-based guideline documents that address various aspects of lung transplantation ranging from candidate evaluation and selection to best practices in peri- and post-transplantation management are needed to optimize survival and quality of life for lung transplant recipients. Additionally, innovative biomarkers that can more accurately detect early allograft dysfunction and allow timely therapeutic intervention to prevent functional decline are needed.

**Table 2.** Key recent advances in lung transplantation.

• Expansion of the lung donor pool (e.g. donation after cardiac death donors) • Advances in the use of extracorporeal life support to bridge candidates to transplant • Development of *ex vivo* lung perfusion • Improved understanding of molecular and cellular mechanisms in primary graft dysfunction • Recognizing interactions of the lung microbiome and recipient immune responses · Disruption of the symbiotic “healthy” lung microbiome · Triggering of dysbiosis by allograft injury, rejection episodes, and immunosuppression • Recognition and characterization of autoimmune responses in allogeneic lung transplantation • Improved understanding of chronic lung allograft dysfunction (CLAD) · Recognition of CLAD’s causes and phenotypes · Role of host–pathogen interactions in CLAD pathogenesis

**Table 3.** Key questions in lung transplantation.

**■ Pre-transplant and transplant surgical procedure issues** • Should frailty be included in determining eligibility and/or computing the lung allocation score? • Should there be an absolute upper age cutoff for eligibility? • How much does body mass index matter? • What is the best approach to screen for/treat gastroesophageal reflux? • How can outcomes for pre-sensitized recipients be optimized? • Who should receive extracorporeal life support (e.g. extracorporeal membrane oxygenation [ECMO]) as a bridge to transplant? • Should *ex vivo* lung perfusion (EVLP) be available to and/or utilized by all transplant centers? • How should EVLP be used to condition marginal donor lungs? • What operation is best for specific disease indications? • Should bronchial arterial circulation be restored when feasible? • Is intra-operative ECMO support superior to cardiopulmonary bypass? • What is the best approach to pre-transplant human leukocyte antigen (HLA) matching? • What is the best approach to performing living-related lung transplants? • When can simultaneous multi-organ transplants (heart–lung, lung–liver, and lung–kidney) be safely performed? **■ Chronic lung allograft dysfunction** • What are the precise roles and mechanisms of alloimmune and autoimmune responses in chronic lung allograft dysfunction (CLAD) pathogenesis? • What is the precise role of antibody-mediated rejection in CLAD onset and progression? • What is the significance of the appearance of *de novo* anti-HLA antibodies in CLAD pathogenesis? • When and how should post-transplant screening and treatment for anti-HLA antibodies be performed? • Can specific biomarkers identify and reliably predict increased risk for the development of CLAD? • Can biomarkers detect the early (subclinical) onset of CLAD and differentiate bronchiolitis obliterans syndrome (BOS) from restrictive allograft syndrome (RAS)? • How can CLAD phenotyping be used to predict prognosis and response to therapy? • What specific agent or combinations of post-transplant immunosuppressive agents are most likely to prevent CLAD and improve allograft and patient survival? • Can early post-transplant therapeutic interventions significantly alter the natural history of CLAD? • When lung retransplantation is performed for end-stage BOS or RAS, what is the risk for the development of significant primary graft dysfunction, acute rejection, and/or CLAD? • Can patients who are more tolerant to their allografts be identified safely and reliably and receive less-intense immunosuppression? • Can induction of tolerance to self-antigens (e.g. collagen V) or strategies to augment regulatory T or B cells to promote and maintain tolerance diminish the risk of CLAD? • Will the use of EVLP techniques to condition the lung allograft diminish the risk of developing CLAD? • What is the best approach for monitoring recipients post-transplant to detect significant occult allograft dysfunction (pulmonary function testing, imaging, or bronchoscopy)? • What is the optimal frequency for obtaining clinical testing to assist in the early detection of CLAD?
